# Longitudinal high-frequency blood biomarkers of axonal injury and astrocytic activation after immune reconstitution in multiple sclerosis

**DOI:** 10.1186/s12974-025-03674-2

**Published:** 2025-12-30

**Authors:** Hernan Inojosa, Luise Werder, Rocco Haase, Undine Proschmann, Pascal Benkert, Jens Kuhle, Hagen B Huttner, Tjalf Ziemssen, Katja Akgün

**Affiliations:** 1 Department of Neurology, Centre of Clinical Neuroscience, University Hospital Carl Gustav Carus Dresden, TUD Dresden University of Technology, Fetscherstraße 74, Dresden, 01307, Germany; 2 Departments of Biomedicine and Clinical Research, Multiple Sclerosis Centre and Research Centre for Clinical Neuroimmunology and Neuroscience (RC2NB), University Hospital and University of Basel, Basel, Switzerland; 3 Department of Neurology, University Hospital and University of Basel, Basel, Switzerland

**Keywords:** Neurofilament light chain, Glial fibrillary acidic protein, Multiple sclerosis, Immune reconstitution therapy, Disease monitoring

## Abstract

**Background:**

Serum neurofilament light chain (sNfL) and glial fibrillary acidic protein (sGFAP) reflect axonal damage and astrocytic injury. Their clinical role in longitudinal real-world monitoring after immune reconstitution therapy (IRT) in multiple sclerosis (MS) remains insufficiently defined. We evaluated longitudinal sNfL and sGFAP dynamics in people with multiple sclerosis (pwMS) treated with alemtuzumab (ATZ) as a model of IRT to determine their prognostic and monitoring value in real-world care.

**Methods:**

PwMS initiating ATZ were prospectively followed up every three months for up to five years. sNfL and sGFAP levels were measured using single molecule array (Simoa) and converted to age- and BMI-adjusted Z scores based on healthy control datasets. Longitudinal trajectories were analysed with generalised linear mixed models adjusted for age, sex, and disease duration. Receiver operating characteristic (ROC) analysis with Youden’s index identified optimal cut-offs for disease activity. Logistic and Cox regression models assessed predictive values. Event-related analyses examined biomarker changes around relapses, MRI activity, progression independent of disease activity (PIRA), and retreatment.

**Results:**

Eighty-four pwMS (mean age 36.5 ± 9.0 years, 76% female) were included. Baseline sNfL Z scores were significantly higher in males and in those with recent MRI activity or treatment failure. sNfL rose transiently one month after the first ATZ course, declined by month 3, and remained stably reduced thereafter. Youden’s index-derived baseline sNfL Z scores ≥ 0.75 modestly predicted disease activity during year 1 (odds ratio: 5.10, 95% CI 1.79–14.49), and Z scores > 1.0 predicted relapses after the first ATZ course (hazard ratio: 2.96, 95% confidence interval 1.36–6.43, *p* = 0.006). Compared to matched pwMS without disease activity, event-related analyses showed significant sNfL elevations around relapses (*p* = 0.004), MRI activity (*p* = 0.015), and retreatment (*p* = 0.002). A transient increase was observed in the 6 months before PIRA events, followed by normalization (*p* = 0.017). sGFAP levels remained overall stable over follow-up (*p* = 0.677) and showed no predictive value.

**Discussion:**

sNfL provided modest predictive and clear monitoring value in pwMS treated with ATZ and may complement individualized follow-up alongside clinical and MRI assessment. In contrast, sGFAP remained overall stable and did not associate with inflammatory events. However, a modest association with subclinical MRI activity was observed, suggesting further research is needed to fully understand the role of sGFAP in monitoring IRT-treated pwMS. These findings support the real-world clinical utility of high-frequency sNfL monitoring for early detection of breakthrough disease activity after IRT.

## Introduction

Predicting and monitoring disease activity remains a major challenge in multiple sclerosis (MS) and other neurological diseases [1]. While clinical examination and neuroimaging remain the mainstays of follow-up, they often fail to capture subclinical activity or to provide timely guidance for treatment decisions. Although several high efficacy disease modifying therapies (DMTs) have been developed with targeted mechanisms of action, the clinical course of people with MS (pwMS) often remains unpredictable, and regular monitoring is essential to guide treatment decisions. Clinical and radiological assessments remain the current standard, but complementary biomarkers may improve disease evaluation and timely treatment decision-taking [2].

In this context, blood biomarkers such as serum neurofilament light chain (sNfL) and glial fibrillary acidic protein (sGFAP) have emerged as promising markers of neuroaxonal and astrocytic damage, respectively [3–9]. While sNfL is increasingly validated as a marker of disease activity, its utility in real-world longitudinal follow-up is still being defined, and evidence for sGFAP is even more limited [10–17]. Because sGFAP reflects astrocytic activation and sNfL reflects axonal injury downstream of inflammatory cascades, jointly tracking them offers a window into neuro-immune dynamics central to MS.

Recently, immune reconstitution therapies (IRT), including alemtuzumab (ATZ), cladribine, and autologous hematopoietic stem cell transplantation (aHSCT), have gained increasing interest as treatment strategies for highly active MS [18, 19]. Although evidence indicates sustained efficacy, repeated ATZ administration may be necessary for pwMS with persistent inflammatory activity. Moreover, most published post-IRT reports rely on annual or sparse sampling, providing only coarse trajectories over time [20–22]. To our knowledge, no short-interval (e.g., 3-monthly) analysis aligned with clinical or radiological events is available. This leaves an important evidence gap on how sNfL and sGFAP should be used after IRT: it is unclear whether serial measurements can both detect re-emerging inflammatory activity after initial suppression and contribute meaningfully to short-term risk stratification in routine care. Complementary biomarkers would therefore help to characterise post-IRT disease course, individualise follow-up and provide actionable signals that support early detection, interpretation, and confirmation of clinical or radiological disease activity.

We aimed to evaluate the longitudinal predictive and monitoring value of sNfL and sGFAP in pwMS undergoing ATZ as a model of IRT. By combining high-frequency biomarker sampling with long-term clinical and MRI follow-up, we sought to determine their utility for individualized disease monitoring in real-world practice as markers of inflammatory and neurodegenerative disease worsening.

## Methods

### Study design

A real-world longitudinal, open-label study was performed. PwMS were consecutively recruited between April 15th, 2010 and June 30th, 2021 from the MS Centre, Centre for Clinical Neurosciences, Department of Neurology, University Hospital Carl Gustav Carus of Dresden, Germany. Inclusion criteria were: (1) age > 18 years; (2) confirmed diagnosis of relapsing-remitting MS according to the 2010 revision of the McDonald criteria; (3) treatment initiation with ATZ according to the standardised infusion scheme as described in the CARE MS I/II clinical trials [21, 22]. PwMS with concomitant neurological diseases that could affect sNfL and sGFAP levels (e.g. ischemic or hemorrhagic stroke, Parkinson’s disease, dementia) were excluded.

### Collection of clinical data and blood samples

Data on time of first diagnosis, previous DMTs or brain MRI characteristics were collected at baseline. Relapse activity and neurological examinations including the Expanded Disability Status Score (EDSS) were assessed prospectively at each visit — during the pre-treatment period, at baseline, and every three months during ATZ treatment. Blood samples for sNfL and sGFAP testing were collected every three months and MRI scans were performed annually starting from baseline.

### Measurement of neural damage biomarkers

Serum or plasma samples were processed immediately after acquisition at the local neuroimmunological lab. Samples were stored at -20 °C until analysis. Measurement of sNfL and sGFAP was performed using single molecule array technology (Simoa Neurology 2-Plex-B Advantage Kits) on a HD-1 instrument (Quanterix, Lexington, MA, USA). Samples were prepared according to the manufacturer’s instructions and were measured in duplicate [23]. Both the intraassay and interassay coefficient of variation were < 10%. Age and body mass index (BMI) adjusted Z scores for sNfL and sGFAP were calculated using a healthy control reference database; sGFAP Z scores were additionally adjusted for sex [8, 24, 25]. Plasma sNfL concentrations were converted to serum as previously described [26].

### Outcomes definitions

- Relapse activity: defined as a new onset or significant worsening of existing neurological symptoms lasting more than 24 h, occurring at least 30 days after a previous relapse, and in absence of fever or infections.
- Progression independent of disease activity (PIRA): defined as an increase in EDSS by at least 1.5 points from a baseline EDSS of 0, by at least 1.0 point from a baseline EDSS of < 5.5, or by at least 0.5 point from a baseline EDSS of ≥ 5.5, and confirmed by persistence in all subsequent evaluations until the first assessment at least six months later. A roving baseline was used with a difference of at least 90 days from previous or subsequent relapses [27].
- MRI worsening: defined as new and/or enlarging T2 lesions compared to the previous MRI and/or gadolinium-enhancing lesions as assessed by an experienced neuroradiologist.
- Retreatment: administration of a third ATZ course due to disease activity according to clinical criteria and product information (at least 12 months after the second ATZ course). Real-world retreatment was not informed by biomarker levels.
- No Evidence of Disease Activity (NEDA > Y1): The treatment goal NEDA, defined as absence of relapses, PIRA, and MRI worsening, persisted after the complete course of ATZ (month 12).
- Evidence of Disease Activity (EDA > Y1): Defined as any relapses, PIRA or MRI worsening after the complete course of ATZ (month 12).

### Statistical analysis

Normal distribution of residuals was inspected using Q-Q plots. Spearman rank correlation coefficients were computed. For the assessment of possible effects of disease duration on the concentrations of sNfL and sGFAP, pwMS were grouped in those with < 7 or ≥ 7 years of disease duration since diagnosis according to the mean of disease duration of the group. The association between biomarker levels and demographic, clinical and imaging parameters was investigated using generalised linear mixed models (GLMM). A gamma distribution with log link function was used because of the right-skewed distribution of the biomarkers. For z-standardised outcomes, a linear link function was used. Separate multivariable models were calculated for each biomarker (sNfL, sGFAP, zNfL, zGFAP) as the dependent variable. Age, sex, body mass index, disease duration, measurement time points, relapse activity in the last 3 months, EDSS and MRI worsening were used as covariates and a random intercept per participant. For longitudinal analyses assessing biomarker changes over time, pairwise comparison between subgroups (i.e. adjusted tests for comparisons between categories of variables) were performed when significant main effects were found. Post hoc pairwise comparisons were Sidak-adjusted for multiple testing. Figure estimates depict model-based estimated marginal means (EMMs) on the response scale obtained by inverse-link back-transformation of the linear predictor. The measurement time points in the observation period after starting ATZ were grouped in quarterly (three-month) intervals for standardised evaluation as these were obtained in a real-world-setting. Although Z scores were also considered, raw values were presented for intra-individual longitudinal analyses, as pwMS served as their own reference group. Chi-square tests were used to compare the proportion of pwMS with elevated biomarkers between baseline and month 18. Receiver operating characteristic (ROC) curves were used to assess the predictive capacity of the biomarkers measured at baseline, month 12, month 18, and month 24 for remaining disease activity-free during the first year, after the first and second ATZ courses. Optimal cut-offs were determined using Youden’s index, and the stability of the area under the curve (AUC) was internally validated using bootstrap resampling. Logistic regression was used to identify factors associated with disease activity and to calculate odds ratios (ORs) for sNfL or sGFAP exceeding significant cut-off values. Cox proportional hazards models were fitted to evaluate the association between baseline and follow-up biomarker levels and subsequent relapse, which represented clearly dated clinical events suitable for time-to-event analysis. Models included prespecified demographic and disease-related covariates.

To explore the association of serum biomarkers with clinical and radiological outcomes, we analysed sNfL and sGFAP concentrations over defined periods surrounding specific events. These events included clinical relapses, PIRA, MRI worsening, or retreatment. For each event, a time window of 6 months (two quarters) before and after the event, starting earliest at 12 months after the second ATZ course, was selected; for MRI the worsening window was 12 months. Comparisons were made against time-matched reference periods from pwMS with sustained no evidence of disease activity (NEDA, no relapses, no MRI activity, no PIRA)-3 status. We defined Month 18 (six months after the second ATZ course) as the ‘biologic re-baseline’ for assessing stable treatment response. The event-related analyses included events occurring earliest at Month 24. This starting point was chosen as it allowed for the 6-month confirmation window required for PIRA detected after the Month 18 re-baseline, and it aligns with the earliest possible time for an ATZ retreatment. For each type of event, only one occurrence per pwMS was included.

*P* values < 0.05 were defined as statistically significant. Model estimates are presented together with the 95% confidence intervals. Statistical analyses were performed using IBM^®^ SPSS^®^ Statistics 30.0.0.0 for Microsoft Windows. GraphPad Prism 5.0 was used for graphical presentation of the results.

### Ethics approval and consent to participate

The study was approved by the Ethics Review Board of the University Hospital of Dresden (EK331112009, EK348092014) and conducted in accordance with the Declaration of Helsinki and Good Clinical Practice guidelines. All participants provided written informed consent prior to inclusion, including consent for blood sampling, biomarker analysis, and linkage with clinical and MRI data. Data were collected as part of the prospective MS cohort registry at the Multiple Sclerosis Centre Dresden.

## Results

### Baseline characteristics

A total of 84 people with MS (pwMS) were included (Table 1). The median EDSS was 3.0 (range: 1.0–8.0) and most participants had at least one relapse or MRI activity within the year preceding treatment initiation. Overall, 29.8% initiated ATZ due to safety concerns with prior therapies.

**Table 1:** Baseline characteristics (*n* = 84)

Parameter	
Age, years, mean (SD)	36.5 (9.0)
Female, *n* (%)	64 (76.2)
BMI, kg/m^2^, mean (SD)	24.8 (5.1)
Disease duration, years, mean (SD)	7.2 (5.7)
< 7 years, *n* (%)	47 (56.0)
≥ 7 years, *n* (%)	37 (44.0)
EDSS score, median (range)	3.0 (2.0–4.0)
Previous DMT, *n* (%)	
None	7 (8.3)
DMT for mild/moderate MS^1^	24 (28.6)
DMT for highly active MS^2^	53 (63.1)
Relapse activity 1 year before ATZ, *n* (%)	
≥ 1 relapse	56 (67.5)
No relapses	27 (32.5)
MRI activity 1 year before ATZ, *n* (%)	
With MRI activity	58 (73.4)
Without MRI activity	21 (26.6)
Reasons for ATZ start, *n* (%)	
Disease activity, treatment naive	7 (8.3)
Disease activity, pre-treatment failure	52 (61.9)
Previous DMT for mild/moderate MS^1^	23 (27.4)
Previous DMT for highly active MS^2^	29 (34.5)
Safety reasons^3^	25 (29.8)
sNfL, median (IQR)	10.7 (7.7–18.8)
sNfL Z score, median (IQR)	1.5 (0.3–2.6)
sGFAP, median (IQR)	78.8 (50.0-107.2)
sGFAP Z score, median (IQR)	0.4 (-0.9–1.4)

*Abbreviations*: *ATZ* alemtuzumab, *BMI* body mass index, *DMT* disease modifying treatment, *EDSS* expanded disability status scale, *IQR* interquartile range, *MRI* magnetic resonance imaging (activity defined as new/enlarged T2- or gadolinium-enhancing lesions), *MS* multiple sclerosis, *SD* standard deviation, *sNfL* serum neurofilament light chain. *sGFAP* serum glial fibrillary acidic protein

^1^Previous immune therapies for mild/moderate MS included interferon beta-1a, glatiramer acetate, dimethyl fumarate, teriflunomide and others (e.g. use of immunoglobulins, secukinumab)

^2^Previous immune therapies for highly active MS included natalizumab, fingolimod and daclizumab

^3^ATZ start due to planned pregnancy in highly active pwMS or increased progressive multifocal leukoencephalopathy risk (e.g. positive John Cunningham virus status and treatment with natalizumab)

### sNfL and sGFAP according to disease characteristics at baseline

Both biomarkers showed a moderate positive correlation (*r* = 0.388, *p* < 0.001). Significantly higher sNfL levels were detected in male pwMS, those with MRI activity during the year prior to ATZ initiation, and in those treated due to active disease compared to individuals switching therapy for safety reasons (Table 2). At baseline, 62.7% of participants exhibited an sNfL Z score > 1.0, which is equivalent to the 84.1st percentile of the healthy reference population (i.e., values higher than those seen in 84% of age- and BMI-adjusted controls). This proportion was notably higher among those with prior disease activity (74.1%) than among those switching due to safety concerns (48%). Furthermore, 33.3% of the cohort had sNfL Z scores > 2.0 (97.7th percentile).

In contrast, sGFAP Z scores showed no significant variation with sex, disease duration, or previous disease activity. At baseline, 34.6% of pwMS had sGFAP Z scores > 1.0, while 10.8% had values > 2.0.

**Table 2:** Biomarkers according to disease characteristics at baseline (*N* = 75)

Parameter	sNfL; mean [95% CI], pg/ml	sNfL Z score; mean [95% CI]	sGFAP; [95% CI], pg/ml	sGFAP Z score; mean [95% CI]
Biomarkers according to sex				
Male	21.4 [14.4–31.9]	1.48 [0.95, 2.00]	84.2 [63.9,110.9]	0.23 [-0.56, 1.03]
Female	12.4 [9.3, 16.7]	1.01 [0.62, 1.39]	74.5 [60.8, 91.3]	-0.11 [-0.70, 0.47]
*p*	**0.025**	**0.032**	0.412	0.419
Biomarkers according to disease duration before ATZ				
< 7 years	13.7 [10.1, 18.4]	1.09 [0.70, 1.49]	69.7 [56.8, 85.7]	0.33 [-0.41, 1.07]
≥ 7 years	19.5 [13.5, 28.3]	1.39 [0.90, 1.88]	89.0 [69.5, 116.4]	-0.21 [-0.81, 0.38]
*p*	0.177	0.242	0.058	0.152
Biomarkers according to relapse activity 1 year before ATZ				
No relapses	16.6 [11.0, 25.2]	1.31 [0.76, 1.85]	71.6 [53.8, 95.3]	-0.20 [-1.02, 0.63]
With relapses	16.0 [11.9, 21.5]	1.17 [0.78, 1.56]	87.6 [71.5, 107.4]	0.32 [-0.27, 0.91]
*p*	0.866	0.657	0.201	0.255
Biomarkers according to brain MRI activity 1 year before ATZ				
No MRI activity	11.2 [7.4, 17.1]	0.50 [0.00, 1.06]	77.2 [57.7, 103.3]	-0.12 [-0.97, 0.73]
With MRI activity	23.7 [18.0, 31.4]	1.98 [1.61, 2.35]	81.3 [67.1, 98.6]	0.24 [-0.031, 0.79]
*p*	**0.001**	**< 0.001**	0.738	0.427
Biomarkers according to indication of ATZ				
Disease activity, no treatment	27.3 [13.5, 55.2]	1.68 [0.75, 2.61]	60.8 [37.4, 98.0]	-0.45 [-1.86, 0.96]
Disease activity pre-treatment on mild/moderate effective DMT^1^	11.8 [7.7, 18.0]	0.65 [0.09, 1.20]^b^	80.7 [60.2, 108.0]	0.06 [-0.78, 0.90]
Disease activity pre-treatment on highly effective DMT^2^	18.9 [12.8, 28.1]	1.81 [1.29, 2.33]^a, c^	87.2 [66.4, 114.5]	0.40 [-0.38, 1.18]
Treatment switch due to safety reasons^3^	11.6 [8.3, 16.2]	0.83 [0.39, 1.27]^b^	92.0 [73.1, 115.8]	0.24 [-0.43, 0.90]
*p*	**0.037**	**0.001**	0.488	0.675

Biomarker values are presented as means with 95% confidence intervals. *P*-values were derived from multivariable generalised linear models with a gamma distribution and log link function, adjusting for age, sex, BMI, disease duration, measurement time points, relapse activity, Expanded Disability Status Scale progression, and MRI worsening. Bold values indicate statistical significance (*p* < 0.05) derived from a generalised linear mixed model with Sidak correction for multiple comparisons

*Abbreviations*: *ATZ* alemtuzumab, *BL* baseline, *CI* confidence interval, *DMT* disease-modifying treatment, *MRI* magnetic resonance imaging (activity defined as new/enlarged T2- or gadolinium-enhancing lesions), *sNfL* serum neurofilament light chain, *sGFAP* serum glial fibrillary acidic protein

^1^Previous immune therapies for mild/moderate MS included interferon beta-1a, glatiramer acetate, dimethyl fumarate, teriflunomide and others

^2^Previous immune therapies for highly active MS included natalizumab, fingolimod and daclizumab

^3^ATZ start due to planned pregnancy in highly active pwMS or increased progressive multifocal leukoencephalopathy risk (e.g. positive John Cunningham virus status and treatment with natalizumab)

^a^*P* < 0.05 compared to Disease activity pre-treatment on mild/moderate effective DMT

^b^*P* < 0.05 compared to Disease activity pre-treatment on highly effective DMT

^c^*P* < 0.05 compared to Treatment switch due to safety reasons

### sNfL and sGFAP levels after ATZ treatment initiation

**Fig. 1:**
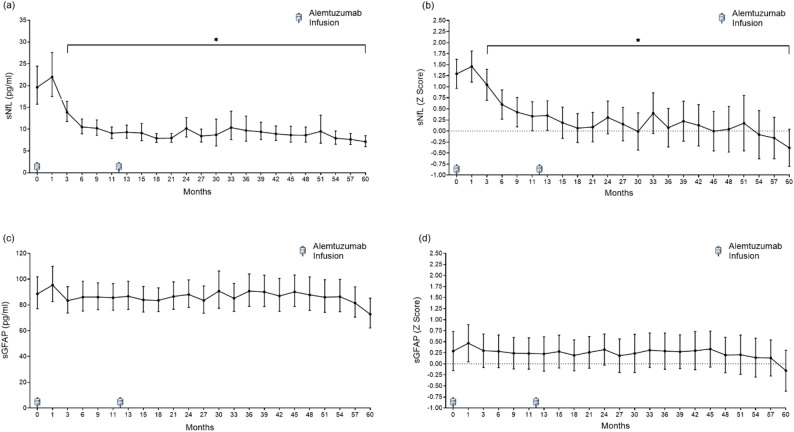
Longitudinal sNfL and sGFAP concentrations following alemtuzumab initiation in pwMS. **a** sNfL and (**c**) sGFAP concentrations over 60 months after treatment initiation. Treatment consisted of infusion courses at month 0 (baseline) and month 12, indicated by infusion markers. Data are presented as mean with 95% confidence intervals. **b**, **d** Age- and BMI-adjusted Z scores based on healthy control reference populations for sNfL and sGFAP, respectively [8, 9, 24]. The dotted line at Z = 0 indicates the average level in healthy controls. Notably, sNfL levels declined after treatment and gradually normalized to healthy control levels. * indicates a *p* < 0.05 compared with baseline, based on a generalised linear mixed model with Sidak correction for multiple comparisons. sNfL: serum neurofilament light chain. sGFAP: serum glial fibrillary acidic protein

The median follow-up duration was 51 months (IQR: 36–63). A transient, non-significant increase in sNfL was observed one month after the first ATZ course, peaking at 22.0 pg/ml (95% CI: 16.8–30.3), corresponding to a mean Z score of 1.46 (95% CI: 1.11–1.81; Fig. 1a–b). Thereafter, sNfL levels declined significantly by month 3 and remained suppressed throughout (all *p* < 0.001 vs. baseline). Notably, no post-treatment sNfL peak was observed after the second course. From month 18 onwards, sNfL Z scores consistently stabilized between 0.0 and 0.5.

sGFAP levels remained unchanged across the entire follow-up, showing minor, non-significant fluctuations (overall time effect: F(22, 1261) = 0.840, *p* = 0.677; Fig. 1c–d).

**Fig. 2:**
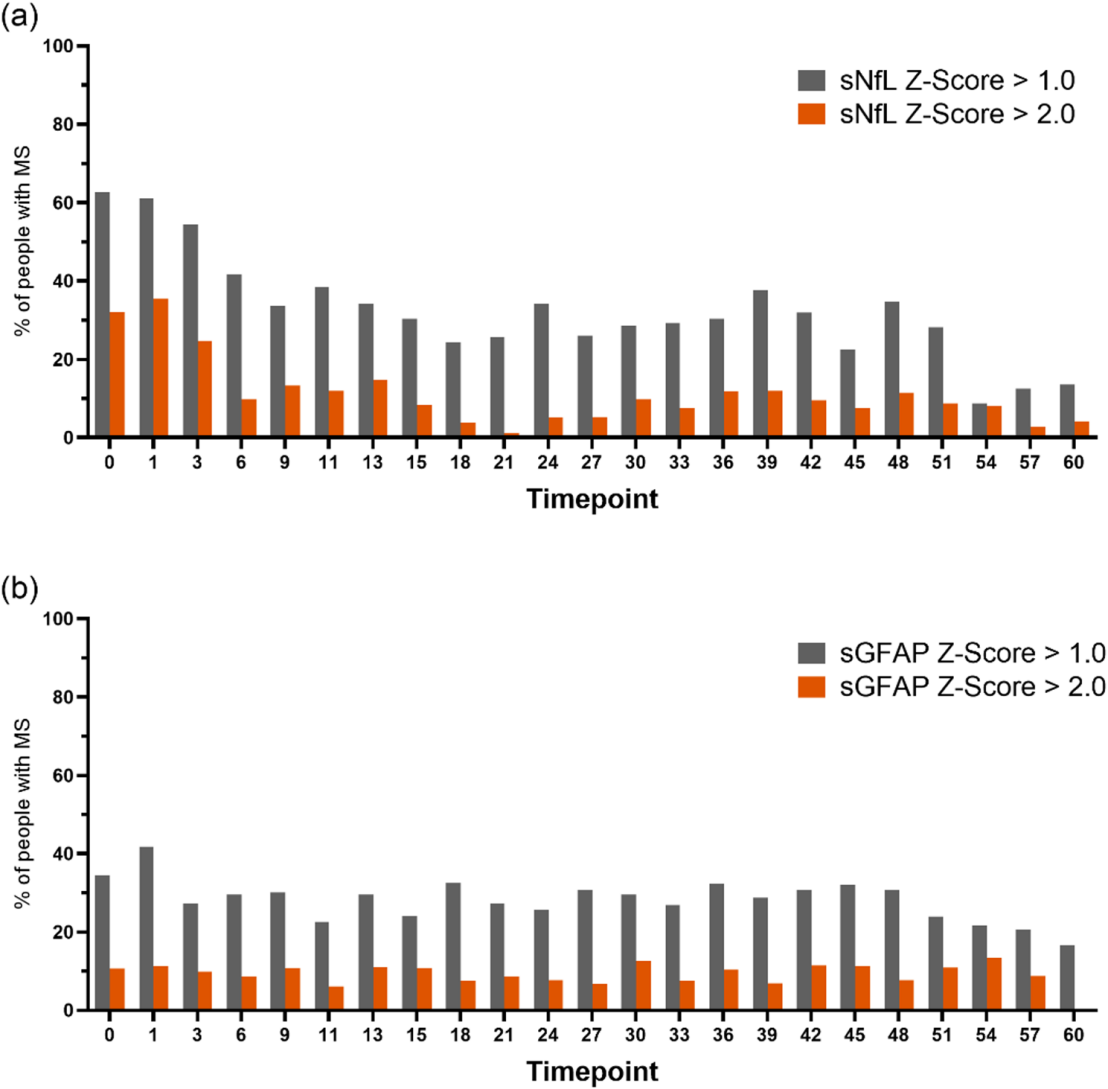
Proportion of pwMS with elevated sNfL and sGFAP Z scores over 60 months following alemtuzumab initiation. **a** Percentage of pwMS with age- and BMI-adjusted sNfL Z scores >1.0 (grey) and >2.0 (orange) at each three-month interval from baseline (month 0) to month 60. **b **Corresponding proportions for sGFAP Z scores. Z scores are derived from healthy reference cohorts adjusted for age and BMI [8, 9, 24]. sNfL: serum neurofilament light chain; sGFAP: serum glial fibrillary acidic protein

Month 18 (six months after the second ATZ course) was set as a biologic re-baseline to assess treatment response. At this point, mean sNfL levels decreased by 11.6 pg/ml (95% CI: −18.2 to − 5.1) compared to baseline. The proportion of pwMS with sNfL Z scores > 1.0 or > 2.0 declined significantly between baseline and month 18 (*p* < 0.001 for both thresholds), as 24.3% had sNfL Z scores > 1.0 and 3.8% >2.0 (Fig. 2). The proportion of pwMS with sGFAP Z scores > 1.0 or > 2.0 remained relatively constant.

### Biomarkers according to clinical hallmarks

To investigate the utility of high-frequency measurement of sNfL and sGFAP as biomarkers of real-time disease activity, biomarker levels were analysed around clinical and radiological events occurring after re-baseline (month 18) compared with age- and sex-matched pwMS with sustained NEDA-3 status.

### Relapses

**Fig. 3:**
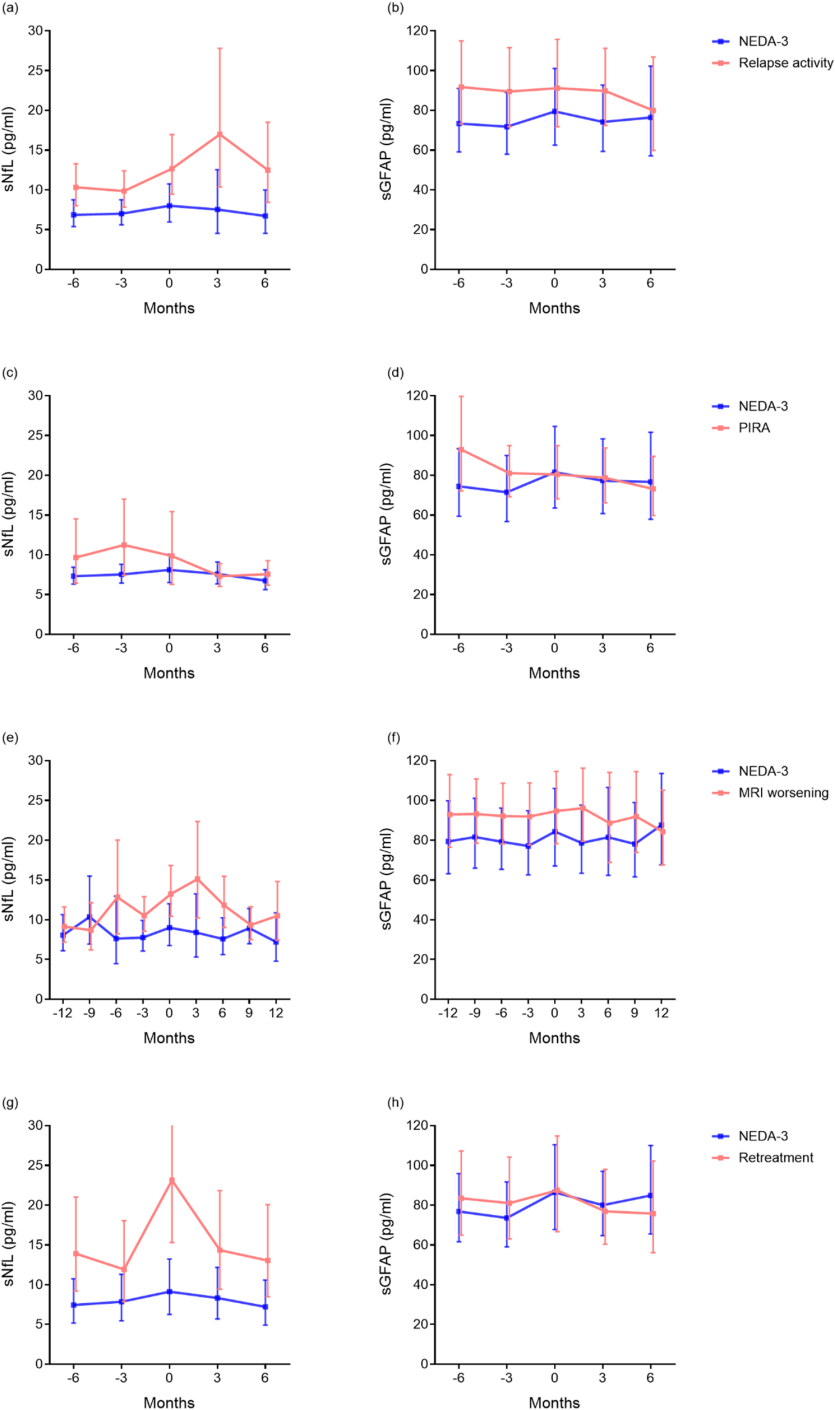
Serum biomarker monitoring at clinical hallmarks. Concentrations of sNfL and sGFAP concentrations (mean and 95% confidence intervals) around upcoming disease activity (red) compared to a matched group of people with MS with NEDA-3 status (blue) in equivalent periods, based on a generalised linear mixed model with Sidak correction for multiple comparisons. Month 0 represents the time point of relapse (a, b), first documented PIRA (c, d), documented MRI worsening (e, f), or retreatment (g,h). A progressive increase of sNfL levels occurs up to 3 months after events with a further decrease afterwards. Concentrations of sGFAP remained relatively stable, although were slightly higher in those with relapses or MRI activity than in the NEDA group. sNfL: serum neurofilament light chain. sGFAP: serum glial fibrillary acidic protein. PIRA: progression independent of disease activity

Following the second ATZ course, 27.2% of pwMS experienced clinical relapses. These individuals exhibited significantly higher sNfL concentrations compared to matched NEDA-3 controls (Table 3). sNfL levels tended to peak within three months after a relapse, followed by a gradual decline (Fig. 3a). sNfL trajectories differed significantly between groups (time x events interaction: F(4,156) = 4.577, *p* = 0.002). In contrast, sNfL levels in the NEDA-3 group remained low and stable throughout. sGFAP concentrations remained stable and showed no significant differences compared to the NEDA-3 group (Fig. 3b; Table 3).

**Table 3:** sNfL and sGFAP levels around upcoming disease activity compared to equivalent periods of NEDA-3 status

Parameter	sNfL, pg/ml, mean [95% CI]	*p*-Values^1^	sGFAP, pg/ml, mean [95% CI]	*p*-Values^1^
NEDA-3 group (*n* = 19)	7.9 [6.4, 9.8]	n/a	84.6 [68.7, 104.2]	n/a
Relapse activity (*n* = 17)	12.2 [9.3, 16.1]	0.004	86.8 [69.0, 109.3]	0.202
MRI worsening (*n* = 26)	11.1 [9.2, 13.2]	0.015	91.7 [85.3, 98.6]	0.424
PIRA (*n* = 41)	9.01 [7.27, 11.16]	0.162	81.01 [69.35, 94.63]	0.657
Retreatment (*n* = 15)	14.8 [10.7, 20.5]	0.002	80.8 [60.0, 103.6]	0.963

Concentrations of sNfL and sGFAP are evaluated in a period of 6 months before and after occurrence of clinical events and compared with a matched reference group with no evidence of disease activity in the same time frame. Values represent the average across all available visits within the ± 6-month window for relapse activity. For MRI worsening, a 12-month window was used. *P*-values were derived from generalised linear mixed models adjusted for age and sex, comparing the event group against the NEDA-3 reference group. NEDA: no evidence of disease activity, MRI: magnetic resonance imaging, EDSS: Expanded Disability Status Scale, sNfL: serum neurofilament light chain, sGFAP: serum glial fibrillary acidic protein^1^*p*-Values from generalised linear mixed models compared with an equivalent sex- and age-matched NEDA-3 group at the visits over the observation period

*Abbreviations*: *sNfL* serum neurofilament light chain, *sGFAP*  serum glial fibrillary acidic protein, *NEDA-3* no evidence of disease activity (i.e. no relapse, MRI activity and PIRA). MRI worsening: new or enlarging T2-hyperintense lesions. PIRA: progression independent of relapse activity

### PIRA

Forty-one PIRA events (present in 45.2% of the cohort) occurred after the second ATZ course. Although overall sNfL levels did not differ significantly between groups across the full observation window (main effect: F(1,183) = 1.97, *p* = 0.162), event-aligned analysis revealed a distinct temporal pattern. sNfL levels showed a tendency to rise in the months preceding PIRA, with visibly higher concentrations at − 6 and − 3 months compared to the NEDA-3 group (Table 3; Fig. 3c). sNfL trajectories differed significantly between groups (time × events interaction: F(4,183) = 3.086, *p* = 0.017), indicating a modest but detectable pre-event increase aligned to PIRA events. In contrast, sGFAP concentrations remained stable and showed no significant differences between groups (Fig. 3d; Table 3).

### MRI activity

MRI-defined disease activity was observed in 34.2% of pwMS after the second ATZ course. PwMS with MRI activity showed significantly higher overall sNfL levels compared to NEDA-3 controls (Table 3). Event-aligned analyses further demonstrated that sNfL trajectories differed significantly between groups (time × events interaction: F(8,329) = 2.747, *p* = 0.006), with a tendency toward higher sNfL levels in the months surrounding imaging-confirmed activity (Fig. 3e; Table 3). In contrast, sGFAP concentrations remained stable and showed no significant differences between MRI-active and NEDA-3 individuals (Fig. 3f; Table 3).

### Retreatment

Fifteen pwMS received a third ATZ course due to recurrent disease activity, with retreatment administered on average 42.1 ± 10.6 months after the initial ATZ infusion (range: 24–70 months). Up to month 18, sNfL levels declined in the retreatment group, and only 4/15 individuals still had sNfL Z scores > 1.0. In the 6-month window surrounding the third ATZ course, retreated pwMS showed significantly higher sNfL levels than matched NEDA-3 controls (Table 3). Event-aligned analyses further demonstrated a distinct temporal pattern, with elevated sNfL values already detectable in the months preceding retreatment and a pronounced peak at the time of retreatment (Fig. 3g). This dynamic was exclusive to the retreatment group, with a significant time × events interaction (F(4,136) = 3.386, *p* = 0.011). In contrast, sGFAP concentrations remained stable and did not differ between retreated and NEDA-3 pwMS (Fig. 3h).

### Biomarkers according to clinical response profiles

**Fig. 4:**
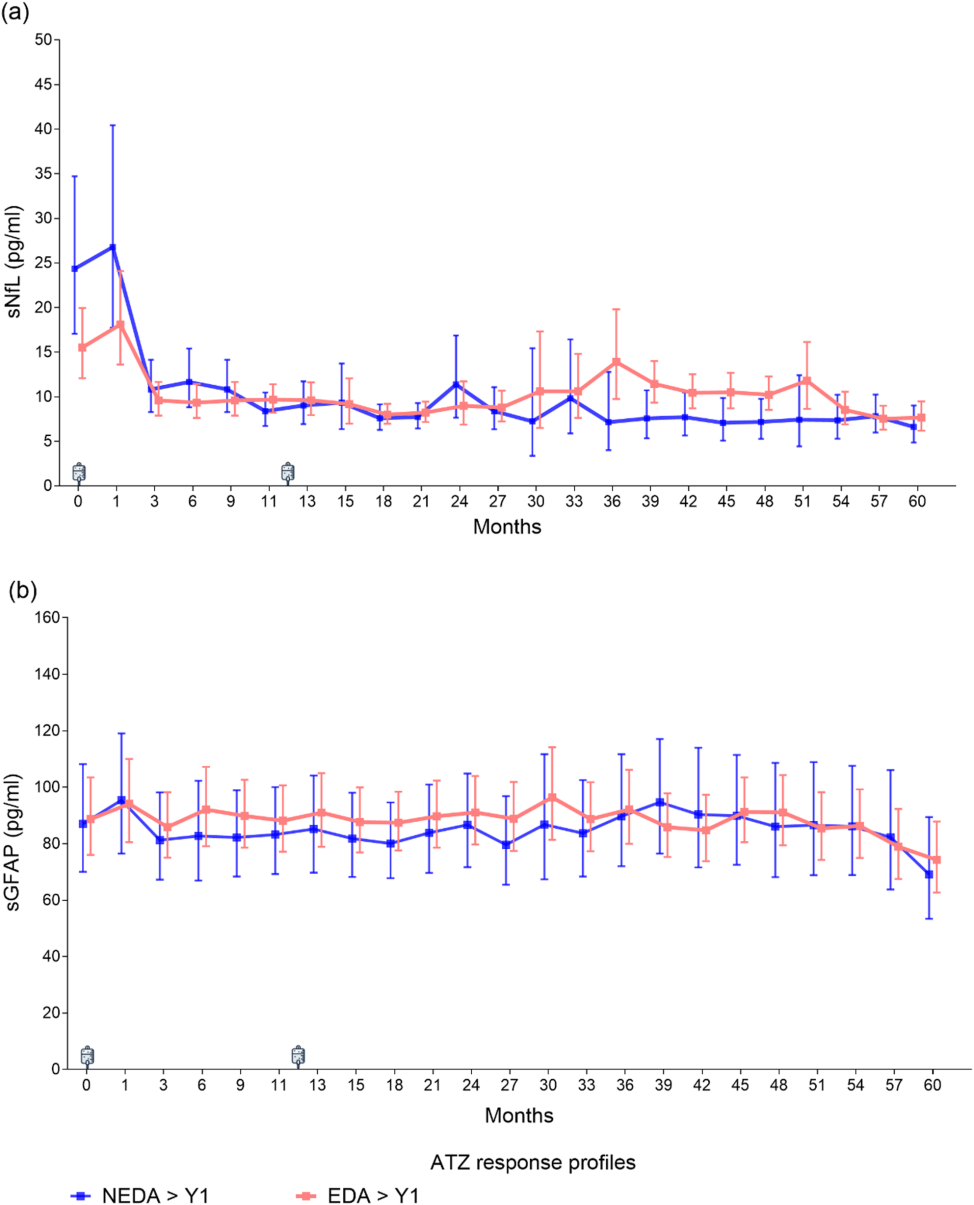
Levels of sNfL and sGFAP in pwMS treated with alemtuzumab (ATZ) grouped according to the evidence of disease activity after complete ATZ administration. ATZ was administered at month 0 (baseline) and month 12. **a** Levels of sNfL were significantly lower at month 3 for both groups (*p* < 0.05) and remained stable for NEDA > Y1 pwMS through the observation period. However, fluctuations are observed in the EDA > Y1 group after month 24. **b** Levels of sGFAP remained stable and relatively similar in both groups. sNfL: serum neurofilament light chain, sGFAP: serum glial fibrillary acidic protein, NEDA > Y1 = no evidence of disease activity after year one and completion of two ATZ courses (blue), EDA > Y1 = evidence of disease activity after year one and completion of two ATZ courses (red)

PwMS with sustained NEDA > Y1 status (*n* = 27; 32.1%) exhibited higher baseline sNfL levels than those with EDA > Y1 (*n* = 57; 67.9%, Fig. 4a). In both groups, sNfL levels transiently increased one month after the first ATZ infusion before declining. From month 3 onward, the NEDA > Y1 group maintained stably low sNfL concentrations, with levels significantly below baseline at all subsequent visits (all *p* < 0.05). In contrast, pwMS with EDA > Y1 demonstrated greater variability over time. Although sNfL remained significantly lower than baseline up to month 24, this suppression was no longer significant between months 30 and 51.

At month 36, 34.2% of the cohort exhibited sNfL Z scores > 1.0, with comparable proportions in the NEDA and EDA groups (31.8% vs. 35.2%). sGFAP concentrations remained stable across both groups throughout follow-up, with no significant differences between NEDA > Y1 and EDA > Y1 subgroups (Fig. 4b).

### sNfL and sGFAP as predictors of disease activity

ROC curve analyses were conducted to assess the prognostic capacity of sNfL and sGFAP at baseline, 12 months, 18 months, and 24 months for predicting future disease activity during year 1, 2, or afterwards. Baseline sNfL Z scores demonstrated modest but statistically significant predictive discrimination for disease activity within the first treatment year (AUC = 0.635, SE = 0.062, 95% CI: 0.513–0.756, *p* = 0.038). The optimal exploratory cut-off identified was an sNfL Z score of 0.75, yielding a sensitivity of 83.3% and a specificity of 49.9% (Youden index = 0.322, OR = 5.10, 95% CI: 1.79–14.49, *p* = 0.002, including 55 pwMS with scores ≥ 0.75, of whom 32 experienced an EDA-event). Raw sNfL levels at Month 12 suggested a discriminatory ability for predicting disease activity during the subsequent treatment year (AUC = 0.643, SE = 0.064, 95% CI: 0.516–0.769, *p* = 0.027). However, an exploratory threshold derived from the ROC curve (8.64 pg/mL) did not retain statistical significance in logistic regression (OR = 0.38, 95% CI: 0.14–1.03, *p* = 0.057), and classification performance was poor.

Cox regression revealed that pwMS with re-baseline sNfL Z scores ≥ 1.0 had an increased risk of relapses after the second ATZ course (HR 2.96, 95% CI: 1.36–6.43, *p* = 0.006, including 52 pwMS with scores ≥ 1.0 and 30 EDA-events). No other Cox models demonstrated significant associations.

## Discussion

This study presented a comprehensive real-world evaluation of sNfL and sGFAP dynamics in a large cohort of highly active pwMS treated with ATZ as an IRT. By combining high-frequency biomarker sampling with long-term follow-up, we were able to characterise biomarker trajectories and their responsiveness to clinical and radiological events.

In this cohort, longitudinal, high-frequency sNfL monitoring predicted and mirrored acute inflammatory disease activity; sGFAP remained stable, consistent with a stronger link to chronic, compartmentalized pathology than to acute focal inflammation. Our findings confirm sNfL as a sensitive biomarker for inflammatory disease activity, while highlighting the relative stability of sGFAP in this highly active cohort after IRT.

At baseline, sNfL levels were elevated compared to reference populations, especially in those with pre-ATZ disease activity, while patients switching for safety reasons had near-normal Z scores, indicating limited ongoing damage [3, 5]. Males showed higher sNfL, suggesting a more aggressive disease course in this group. Baseline sGFAP values were generally low, with higher absolute concentrations in those with longer disease duration, although Z scores remained unchanged after adjustment, in line with previous mixed reports on GFAP and disease duration [4, 6, 28].

Nearly two-thirds of pwMS had sNfL Z > 1.0 at baseline, while sGFAP Z scores remained largely normal, underscoring their distinct biology: sNfL reflects acute inflammation and axonal loss, making it a sensitive marker of acute tissue damage in relapsing remitting MS [10, 16, 29–33], whereas sGFAP reflects astrocytic activation, often elevated in progressive disease but less sensitive to acute activity in inflammatory cohorts [6, 9, 34, 35].

During treatment, sNfL dropped substantially after ATZ initiation and remained low, consistent with CARE-MS I trial data and other IRT [11, 12, 14, 15, 17, 36]. The reduction was greatest in those with highest baseline levels (~ 50% decline within six months) underscoring ATZ’s efficacy in suppressing inflammatory activity [11]. Interestingly, high frequency assessments revealed a transient sNfL increase one month after the first course of ATZ, especially in those treated for active disease. Similar patterns were reported after aHSCT [19]. However, the underlying mechanism remains unclear, as direct cytotoxic effects are unlikely with ATZ. This transient sNfL elevation may reflect residual disease activity in the initial weeks post-ATZ rather than an indicator of treatment efficacy or adverse effects. A mixture of residual inflammatory activity and peri-infusion factors, including high-dose corticosteroid premedication, possible early infections, and infusion reactions can also contribute.

In line with prior work on induction therapies, similar post-treatment declines in sNfL have been reported for cladribine, another IRT, increasingly used due to safety concerns associated with alemtuzumab that induces transient lymphocyte depletion followed by immune renewal [37–39]. However, to our knowledge, previous cladribine studies are based on smaller cohorts with annual or semi-annual sampling, providing only coarse trajectories. Whether sNfL rises again in association with breakthrough activity after initial suppression thus remains in that context unknown.

Importantly, while elevated sNfL during acute disease activity is well documented [10, 16, 29, 40, 41], detailed characterizations of biomarker trajectories after IRT are scarce and often based on small cohorts. Moreover, to our knowledge, no prior study has examined short-interval sNfL dynamics or event-aligned biomarker changes around clinical and radiological events. In our analysis, sNfL increased gradually in the months preceding relapses, MRI worsening, or retreatment, highlighting its sensitivity to breakthrough inflammatory activity under ATZ.

In contrast, the overall stability of sGFAP is consistent with prior ATZ-treated cohorts [11, 12, 16, 17], although the role of sGFAP as a marker for treatment response in highly inflammatory cohorts remains unclear [42]. The relative flatness of sGFAP despite events is biologically plausible, as sGFAP reflects astrocytic activation and tissue remodeling, which may preferentially track compartmentalized, smoldering inflammation rather than brief, focal inflammatory events [4, 34, 42, 43]. After IRT, residual inflammatory activity seems to be focal, which may generate limited astrocytic damage [18, 19, 44]. However, its stability could reflect a positive response, indicating absent astrocytic activation despite ongoing inflammation.

Evidence from other IRT cohorts is still limited as most published series are based on smaller samples or fewer predefined sampling time points rather than high-frequency follow-up. As a result, detailed event-aligned modelling comparable to our approach is limited in those datasets, but their aggregate trajectories are broadly consistent with a largely stable sGFAP [14, 17, 19]. Moreover, our results contrast with a B-cell-depleted cohort, where sGFAP increased over time and correlated with PIRA events [9]. This may reflect different biological pathways or patient selection, as B-cell-depletion and IRT are typically used in distinct clinical contexts.

Considering the predictive value of the biomarkers, two discrete but modest sNfL signals emerged across the treatment course. Baseline sNfL Z scores demonstrated limited but statistically significant discrimination for identifying pwMS who developed inflammatory activity during the first treatment year, and an exploratory threshold (Z ≥ 0.75) emerged. In addition, raw sNfL concentrations at month 12 showed a similar association with disease activity during the subsequent year, although a corresponding dichotomised cut-off did not retain statistical significance and classification performance was poor, underscoring that these findings reflect weak discriminatory tendencies rather than clinically validated thresholds. Beyond these signals, only one further association was observed: pwMS with re-baseline sNfL Z scores ≥ 1.0 had a higher risk of relapse after the second ATZ course. By contrast, sGFAP showed no predictive value for clinical relapses, PIRA or MRI activity at any examined time point.

Together, these results suggest that while sNfL carries some prognostic information (particularly at treatment initiation and during early follow-up), its predictive performance remains limited, and sGFAP does not appear useful for forecasting near-term disease activity. Importantly, these isolated predictive signals contrast with the clearer temporal patterns revealed through high-frequency longitudinal sampling. While static measurements offered only modest prognostic information, dynamic changes in sNfL aligned more consistently with clinical and MRI activity, and the need for retreatment. In line with this, the longitudinal contrasts suggest a divergence in sNfL recovery profiles between response groups: pwMS with sustained NEDA > Y1 maintained suppressed sNfL levels throughout follow-up, whereas those with EDA > Y1 showed increasing variability and, between Months 30 and 51, no longer exhibited significant suppression at group-level relative to baseline. However, follow-up times may have limited our ability to confirm the persistence and magnitude of this divergence. This pattern may also suggest that loss of sustained sNfL reduction after the initial post-IRT decline may signal re-emerging inflammatory activity or accumulating neuroaxonal injury despite prior immune reconstitution.

A striking finding of our study was the high rate of PIRA, which occurred in 45.2% of our cohort despite ATZ treatment. This figure is high, but must be interpreted in the context of our cohort’s characteristics: a mean disease duration of 7.2 years, a median baseline EDSS of 3.0, and a high history of treatment failure. Interestingly, pwMS with PIRA events had slightly elevated sNfL levels compared to NEDA-3 counterparts, especially before the events. This finding suggests that these events may be associated with preceding increased inflammatory activity that is not fully suppressed by IRT.

Certain limitations must be considered. Although relatively large for a monocentric real-world study, sample size and follow-up limited subgroup analyses, particularly for sGFAP and disability progression. Typical challenges of real-world data—patient dropouts, missing values, and heterogeneous follow-up durations introduced variability and reduced consistency compared to controlled clinical trials. Clinical assessments and MRI evaluations were not uniformly scheduled, although we applied standardised quarterly intervals to improve comparability. Furthermore, the monocentric design and tertiary-care setting may have introduced selection bias, likely overrepresenting individuals with higher disease activity and closer monitoring. However, the consistent trends observed in sNfL and sGFAP trajectories, along with relatively narrow confidence intervals, support the robustness of our findings despite these constraints. Still, the single-centre nature of the study limits the generalisability of results to broader MS populations. Lastly, while serum-based biomarkers such as sNfL and sGFAP may support disease monitoring, their implementation in routine practice faces technical challenges—particularly regarding assay standardisation and inter-laboratory comparability. We did not define what constitutes a clinically relevant sNfL rise after Month 18; establishing validated, age-adjusted thresholds and confirmation rules should be the focus of future prospective work.

In conclusion, this real-world study highlights the practical value of sNfL as a dynamic and clinically relevant biomarker for monitoring inflammatory disease activity in pwMS receiving IRT with ATZ. By contrast, sGFAP remained stable and did not reflect relapses or MRI activity, underscoring differences in biomarker biology and their potential complementary use. Integration of sNfL with routine clinical and radiological assessments may inform treatment evaluation and support timely detection of breakthrough inflammatory activity. Beyond MS, these findings illustrate how blood biomarkers can contribute to biomarker-guided monitoring strategies across neurological diseases. Future work should extend follow-up, assess diverse MS phenotypes, and test multimodal biomarker panels to capture smoldering disease courses. In particular, prospective studies should evaluate whether a post–Month-18 rise in sNfL (e.g. relative to the Month-18 biologic re-baseline) adds actionable value for individualized retreatment decisions. As clinically relevant ‘rise’ thresholds and confirmation rules for sNfL are not yet established, these studies should define cut-points, repeat-sampling requirements, and decision-analytic endpoints.

## Data Availability

The datasets generated and/or analysed during the current study are not publicly available due to local and institutional data-protection policies. Deidentified data will be made available by the corresponding author(s) upon reasonable request.
